# The positive effect of dupilumab on comorbid asthma in patients with atopic dermatitis

**DOI:** 10.1002/clt2.12219

**Published:** 2023-01-16

**Authors:** Lotte S. Spekhorst, Marlies de Graaf, Lisa P. van der Rijst, Nicolaas P. A. Zuithoff, René C. Schweizer, Marijke Kamsteeg, Inge Haeck, Anneke M. T. van Lynden‐van Nes, Paula van Lumig, Geertruida L. E. Romeijn, Marie‐Louise Schuttelaar, Marjolein S. de Bruin‐Weller

**Affiliations:** ^1^ Department of Dermatology and Allergology National Expertise Center for Atopic Dermatitis University Medical Center Utrecht Utrecht The Netherlands; ^2^ Julius Center for Health Sciences and Primary Care University Medical Center Utrecht Utrecht The Netherlands; ^3^ Department of Pulmonology University Medical Center Utrecht Utrecht The Netherlands; ^4^ Department of Dermatology Radboud University Medical Center Nijmegen The Netherlands; ^5^ Department of Dermatology Reinier de Graaf Gasthuis Delft The Netherlands; ^6^ Department of Dermatology Meander Medisch Centrum Amersfoort The Netherlands; ^7^ Department of Dermatology University Medical Center Maastricht Maastricht The Netherlands; ^8^ Department of Dermatology University Medical Center Groningen Groningen The Netherlands

To the Editor,

IL‐4 and IL‐13 are Type‐2 (T2) inflammatory cytokines and key drivers in T2 immune response, which is considered to play a central role in the pathogenesis of several atopic diseases such as atopic dermatitis (AD) and asthma. Dupilumab, a fully human monoclonal antibody, binds to the α‐subunit of the interleukin (IL)‐4 receptor and blocks the signaling pathway of IL‐4 and IL‐13.^1^ It is the first antibody‐based treatment that became available for the treatment of AD and is also registered for severe T2 asthma.^2^ Several studies reported improved clinical outcomes and sustained reduction of T2 inflammatory biomarkers for AD as well as asthma by using dupilumab.^3^, ^4^ Since the majority of AD patients have comorbid asthma,^5^ the aim of this study was to investigate the effect of dupilumab on asthma in patients treated for AD with dupilumab in daily practice.

This study consecutively included adult AD patients with comorbid asthma and at least one measurement of the Asthma Control Questionnaire (ACQ)‐5 (scale 0–6), and/or FEV1, who started dupilumab treatment for AD and participated in the BioDay registry from October 2017 to June 2022. The ACQ‐5 was used as patient‐reported outcome, consisting five questions on symptom control of asthma. Following The Global Initiative for Asthma (GINA)‐guidelines controlled asthma in a real‐life setting is defined as ACQ‐5 < 0.5.^6^ In a subset of patients using inhaled steroids regularly, Forced Expiratory Volume in 1 s (FEV1) was assessed, partially combined with Fractional exhaled Nitric Oxide (FeNO). Levels of NO are increased in the exhaled breath of patients with T2 asthma and provide an objective biomarker of airway inflammation, with the following cut‐off points: <25 parts per billion (ppb) (low), 25–50 ppb (intermediate), ≥50 ppb (high).^7^ Primary effectiveness endpoints were the mean change from baseline in ACQ‐5 and FEV1 at weeks 16 and 52. Secondary effectiveness endpoints were: ACQ‐5 < 0.5, FEV1 ≥ 80% predicted, and FeNO at weeks 16 and 52. For the analysis of continuous outcomes, a mixed model with a random intercept was used and results were used to estimate means with 95% confidence intervals (CI). Continuous variable FeNO, with a highly skewed distribution, was log‐transformed. These estimated mean log‐transformed were transformed back to median FeNO values (with 95% CIs). Descriptive analysis was used for the categorical endpoints. The role of T2‐indicator blood eosinophilia (>0.4 × 10*9/L) at the start of dupilumab treatment on the primary endpoint FEV1 is shown in the Appendix Table 3.

A total of 304 AD patients treated with dupilumab and comorbid asthma with an ACQ‐5 and/or FEV1 measurement were included (see Appendix Table 1 for the baseline characteristics per cohort). All primary effectiveness endpoints significantly improved after 16‐ and 52 weeks of dupilumab treatment compared to baseline (see Figure 1 and Appendix Table 2). Mean ACQ‐5 was 1.32 (95% CI 1.20–1.45; *n* = 236) at baseline, and significantly improved over time (*p* < 0.00), with −0.24 (95% CI −0.38 to −0.10; *n* = 173) at week 16 and −0.26 (95% CI −0.43 to −0.09; *n* = 110) at week 52. Mean FEV1 at start of treatment was 2.96 L (95% CI 2.79–3.13; *n* = 104) and significantly improved over time (*p* < 0.00) with 0.10 L (95% CI 0.03–0.16) and 0.12 L (95% CI 0.05–0.19) at week 16 (*n* = 81) and 52 (*n* = 64), respectively. No significant change for ACQ‐5 and FEV1 was found between week 16 and 52. Secondary effectiveness endpoints are presented in Figure 1 and Appendix Table 2. At start of dupilumab treatment median FeNO (*n* = 22) was 23.43 ppb (95% CI 16.37–33.53) and significantly decreased over time (*p* < 0.00), to 13.13 ppb (95% CI 10.49–16.45; *n* = 17) at 16‐weeks and to 15.24 ppb (95% CI 12.38–18.76; *n* = 21) at 52‐weeks of treatment (Figure 1). At start of treatment, 20.8% and 58.7% of the patients had an ACQ‐5 <0.5 and FEV1 ≥ 80% and increased to 28.2% and 68.8% after 1 year of treatment, respectively (Appendix Table 2).

**FIGURE 1:**
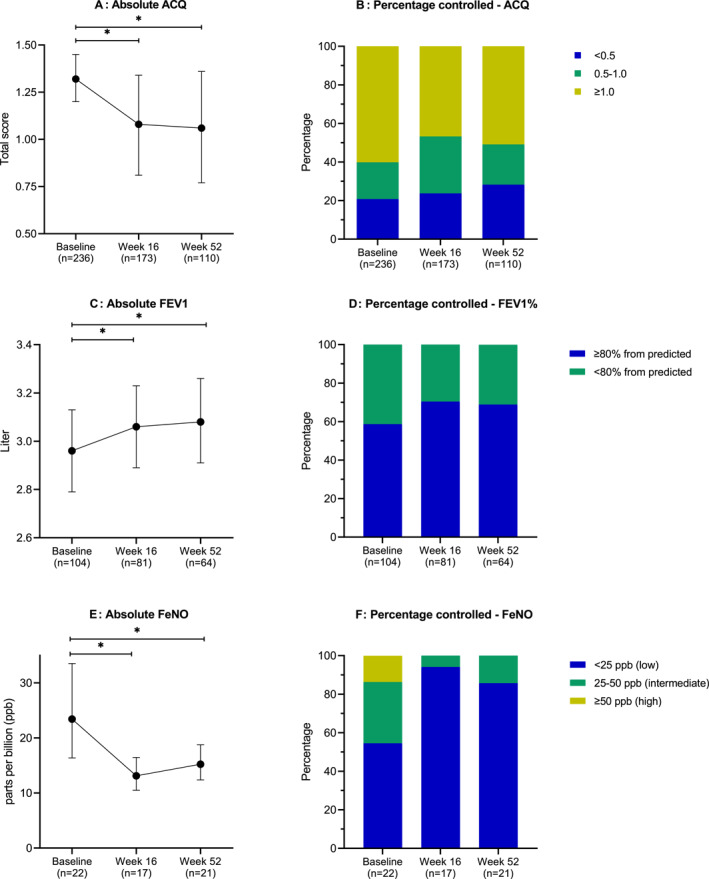
Effectiveness outcomes for asthma status during 1 year of dupilumab treatment in AD patients with comorbid asthma. (A) Absolute change in ACQ. Bars represent mean and 95% CI, (B) Percentage controlled ACQ‐5 based on cut‐off points; (C) Absolute change in FEV1 in L. Bars represent mean and 95% CI, (D) Percentage controlled FEV1% from predicted; (E) Absolute change in FeNO per ppb. Bars represent median and 95% CI, (F) Percentage controlled FeNO based on cut‐off points. ACQ‐5, Asthma Control Questionnaire; CI, Confidence Interval; FEV1, Forced Expiratory Volume in 1 s; FeNO, Fractional exhaled Nitric Oxide; Ppb, parts per billion. *p*‐values based on overall likelihood ratio tests for time. *p < 0.05.

In contrast to dupilumab studies, primarily focused on severe uncontrolled asthma, the majority of the patients in our study had relatively mild asthma with a mean ACQ‐5 of 1.32 and FEV1 of 2.96 L at start of treatment. Nevertheless, ACQ‐5, FEV1 and FeNO significantly improved already after 16 weeks. Previous randomized controlled trials (RCTs) investigating the efficacy and safety of dupilumab in patients with uncontrolled asthma showed a more impressive effect on the primary endpoints.^3^ However, similar substantial improvements are difficult to achieve in our AD patients with relatively mild asthma.

As shown in the Appendix, no profound effect of T2‐indicator, blood eosinophilia, at the start of treatment was found regarding the effectiveness of dupilumab on FEV1. On the contrary, in the asthma studies,^3^ the most robust results were observed in patients with elevated T2‐indicators, including eosinophil counts. Possibly the effect of dupilumab is less dependent on T2‐indicator blood eosinophilia in patients with mild asthma.

A limitation of the study is the missing data due to the daily practice setting and COVID‐pandemic. Additionally, spirometry measurements were only conducted in patients using inhaled corticosteroids thereby excluding patients with mild asthma.

In conclusion, 1 year of dupilumab treatment primarily indicated for AD resulted in a significant improvement of comorbid asthma with the largest effect in the first 16 weeks. Dupilumab treatment in AD patients provides an additional advantage for patients with comorbid asthma.

## AUTHOR CONTRIBUTIONS

All authors have made substantial contributions to conception and design of this study and have been involved in drafting or revising the manuscript. All authors have given final approval of the version to be published an agreed to be accountable for all aspects of the work. Lotte S. Spekhorst had full access to all the data in the study and takes responsibility for the integrity of the data and the accuracy of the data analysis.

## CONFLICT OF INTEREST

Lotte S. Spekhorst is a speaker for Abbvie. Marlies de Graaf is an advisor, consultant, speaker or investigator for Sanofi‐Genzyme, Regeneron Pharmaceuticals, LEO Pharma and Eli Lilly. Lisa P. van der Rijst has nothing to disclose. Nicolaas P. A. Zuithoff has nothing to disclose. René C. Schweizer has nothing to disclose. Marijke Kamsteeg has nothing to disclose. Inge Haeck is an advisor, consultant, speaker or investigator for Sanofi‐Genzyme, LEO pharma, AbbVie and Eli Lilly. Anneke M. T. van Lynden‐van Nes has nothing to disclose. Paula van Lumig has nothing to disclose. Geertruida L. E. Romeijn has nothing to disclose. Marie‐Louise Schuttelaar is an advisor, consultant, speaker and/or investigator for AbbVie, Pfizer, LEO Pharma, Regeneron, Sanofi Genzyme, Eli Lilly and Galderma. She has received grants from Regeneron, Sanofi Genzyme, Novartis and Pfizer. Marjolein S. de Bruin‐Weller has been a consultant, advisory board member, and/or speaker for AbbVie, Almirall, Arena, Aslan, Eli Lilly, Galderma, Janssen, Leo Pharma, Pfizer, Regeneron, and Sanofi‐Genzyme.

## FUNDING INFORMATION

Regeneron Pharmaceuticals; AbbVie; Sanofi; LEO Pharma; Eli Lilly and Company; Pfizer.

## Supporting information

Supplementary Material S1
