# Supplementary material for: The positive effect of dupilumab on comorbid asthma in patients with atopic dermatitis
Source: Clin Transl Allergy. 2023 Jan 16;13(1):e12219. doi: 10.1002/clt2.12219 (PMC9842782; doi:10.1002/clt2.12219)
Supplement: Supplementary file 1 — Supplementary Material S1 [file CLT2-13-e12219-s001.docx]

**Appendix**

**Eosinophilia at the start of dupilumab treatment**

A post-hoc analysis was performed to assess the role of T2-indicator blood eosinophilia (>0.4x10*9/L) at start of treatment on the effectiveness of dupilumab on the primary endpoint Forced Expiratory Volume in 1 second (FEV1). No significant differences (p=0.87) in treatment benefit was observed in patients with or without eosinophilia at start of treatment (Table E2), with differences smaller than 0.02 L. However, patients with blood eosinophilia at baseline had a higher FEV1 at start of treatment (3.13 L (2.91-3.35)) compared to patients without blood eosinophilia (2.78 L (95% CI 2.54-3.02)).

**Table 1.** Patient and baseline characteristics for AD patients with comorbid asthma treated with dupilumab.

|  | ACQ cohort | FEV1 cohort | FeNO cohort |
| --- | --- | --- | --- |
| N (%) | 286 (100.0) | 116 (100.0) | 39 (100.0) |
| Gender (Male), n (%) | 147 (51.4) | 61 (52.6) | 17 (43.6) |
| Age, mean (SD) | 41.3 (15.6) | 42.2(15.2) | 45.1 (14.8) |
| BMI, mean (SD) | 26.3 (5.1) | 26.9 (5.2) | 26.6 (4.5) |
| Missing | 76 | 29 | 3 |
| Age at AD onset, n (%) |  |  |  |
| Childhood | 263 (92.9) | 110 (94.8) | 37 (94.9) |
| Adolescence | 8 (2.8) | 3 (2.6) | 2 (5.1) |
| Adulthood | 12 (4.2) | 3 (2.6) | 0 (0) |
| Missing | 3 | 0 | 0 |
| Use of inhalant corticosteroids, n (%) | 201 (72.0) | 116 (100) | 39 (100) |
| Missing | 7 | 0 | 0 |
| Atopic comorbid |  |  |  |
| Allergic rhinitis, n (%) | 227 (79.9) | 93 (80.2) | 29 (74.4) |
| Missing | 2 | 0 | 0 |
| Allergic conjunctivitis, n (%) | 196 (69.8) | 89 (77.4) | 31 (79.5) |
| Missing | 5 | 1 | 0 |
| Food allergy, n (%) | 174 (61.7) | 75 (65.2) | 27 (69.2) |
| Missing | 4 | 1 | 0 |
| EASI score, mean (SD) | 16.9 (14.4) | 18.6 (11.0) | 16.7 (8.8) |
| Missing | 1 | 0 | 0 |
| IGA score, median (IQR) | 3.0 (3.0-4.0) | 3.0 (3.0-4.0) | 3.0 (3.0-4.0) |
| Missing | 3 | 0 | 0 |
| Eosinophils levels (x10*9/L), median (IQR) | 0.4 (0.2-0.6) | 0.5 (0.2-0.6) | 0.5 (0.2-0.6) |
| Missing | 26 | 5 | 3 |
| Eosinophilia (>0.4x10*9/L), n (%) | 122 (46.9) | 62 (55.9) | 21 (58.3) |
| Missing | 26 | 5 | 3 |

BMI, body mass index; CI, Confidence Interval; EASI, Eczema Area and Severity Index; IGA, Investigator Global Assessment Scale; IQR, Interquartile range; SD, Standard Deviation.

**Table 2.** Primary and secondary outcomes for asthma status during dupilumab treatment in atopic dermatitis patients.

|  | **Baseline** | **Week 16** | **Week 52** | ***p*-value^1^** |
| --- | --- | --- | --- | --- |
| ACQ-5 score, n | 236 | 173 | 110 | n.a. |
| Spirometry, n | 104 | 81 | 64 | n.a. |
| FeNO, n | 22 | 17 | 21 | n.a. |
|  |  |  |  |  |
| **Primary endpoints^2^** |  |  |  |  |
| ACQ-5, mean (95% CI) | 1.32 (1.20-1.45) | 1.08 (0.81-1.34) | 1.06 (0.77-1.36) | <0.00 |
| FEV1, mean (95% CI) | 2.96 (2.79-3.13) | 3.06 (2.89-3.23) | 3.08 (2.91-3.26) | <0.00 |
|  |  |  |  |  |
| **Secondary endpoints^3^** |  |  |  |  |
| FeNO, median (95% CI)^2^ | 23.43 (16.37-33.53) | 13.13 (10.49-16.45) | 15.24 (12.38-18.76) | <0.00 |
| FeNO, <25 ppb, n(%) | 12 (54.5) | 16 (94.1) | 18 (85.7) | n.a. |
| FeNO, 25-50 ppb, n(%) | 7 (31.8) | 1 (5.9) | 3 (14.3) | n.a. |
| FeNO, ≥50, n(%) | 3 (13.6) | 0 (0) | 0 (0) | n.a. |
|  |  |  |  |  |
| ACQ-5 ≥1.0, n (%) | 142 (60.2) | 81 (46.8) | 56 (50.9) | n.a. |
| ACQ-5 <0.5, n (%) | 49 (20.8) | 41 (23.7) | 31 (28.2) | n.a. |
| FEV1 (% from predicted) ≥ 80, n (%) | 61 (58.7) | 57 (70.4) | 44 (68.8) | n.a. |

^1^*P*-values based on overall likelihood ratio tests for time. ^2^A mixed model with a random intercept was used, results were used to estimate means with 95% confidence intervals. Continuous variable FeNO, with a highly skewed distribution, was log-transformed. These estimated mean log-transformed were transformed back to median FeNO values (with 95% CIs). ^3^Descriptive analysis was used for the categorical endpoints.

ACQ-5, Asthma Control Questionnaire; CI, Confidence Interval; FEV1, Forced Expiratory Volume in 1 second; FeNO, Fractional exhaled Nitric Oxide; IQR, Interquartile range; n.a., not applicable; Ppb, parts per billion; SD, Standard Deviation.

**Table 3.** The role of T2-indicator, blood eosinophilia, at start of treatment on the effectiveness of dupilumab on the primary endpoint FEV1.

|  | **Baseline FEV1** | **Week 16 FEV1** | **Week 52 FEV1** | ***p*-value** | |
| --- | --- | --- | --- | --- | --- |
| Eosinophils levels  >0.4 x10*9/L,  mean (95% CI) | 3.13 (2.90-3.36) | 3.22 (2.99-3.45) | 3.26 (3.02-3.49) | 0.87 |  |
| Eosinophils levels  ≤0.4 x10*9/L,  mean (95% CI) | 2.76 (2.50-3.02) | 2.85 (2.60-3.11) | 2.89 (2.63-3.15) |  |  |

^a^Eosinophilia >0.4 x10*9/L. CI, Confidence interval. ^1^*P*-values based on overall likelihood ratio tests for time by blood eosinophilia.
